# Left Ventricular Function after Arterial Switch Operation as Assessed by Two-Dimensional Speckle-Tracking Echocardiography in Patients with Simple Transposition of the Great Arteries

**Published:** 2016-07-06

**Authors:** Elaheh Malakan Rad, Yazdan Ghandi, Armen Kocharian, Mohammadreza Mirzaaghayan

**Affiliations:** 1 *Children’s Medical Center, Tehran University of Medical Sciences, Tehran, Iran.*; 2 *Amirkabir Hospital, Arak University of Medical Sciences, Arak, Iran.*

**Keywords:** *Arterial switch operation*, *Echocardiography*, *Transposition of great vessels*

## Abstract

**Background:** The late postoperative course for children with transposition of the great arteries (TGA) with an intact ventricular septum (IVS) is very important because the coronary arteries may be at risk of damage during arterial switch operation (ASO). We sought to investigate left ventricular function in patients with TGA/IVS by echocardiography.

**Methods:** From March 2011 to December 2012, totally 20 infants (12 males and 8 females) with TGA/IVS were evaluated via 2-dimensional speckle-tracking echocardiography (2D STE) more than 6 months after they underwent ASO. A control group of age-matched infants and children was also studied. Left ventricular longitudinal strain (S), strain rate (SR), time to peak systolic longitudinal strain (TPS), and time to peak systolic longitudinal strain rate (TPSR) were measured and compared between the 2 groups.

**Results:** Mean ± SD of age at the time of study in the patients with TGA/IVS was 15 ± 5 months, and also age at the time of ASO was 12 ± 3 days. Weight was 3.13 ± 0.07 kg at birth and 8.83 ± 1.57 kg at the time of ASO. Global strain (S), Time to peak strain rate (TPSR), and Time to peak strain (TPS) were not significantly different between the 2 groups, whereas global strain rate (SR) was significantly different (p value < 0.001). In the 3-chamber view, the values of S in the lateral, septal, inferior, and anteroseptal walls were significantly different between the 2 groups (p value < 0.001), and SR in the posterior wall was significantly different between the 2 groups (p value < 0.001). There were no positive correlations between S and SR in terms of the variables of heart rate, total cardiopulmonary bypass time, and aortic cross-clamp time. There were no statistically significant differences between the 2 groups regarding S, SR, TPS, and TPSR in the anteroseptal and posterior walls in the 3-chamber view and in the lateral and septal walls in the 4-chamber view.

**Conclusion:** We showed that between 6 and 18 months after a successful ASO, the parameters of S, SR, and global TPS were normal in our patients with TGA/IVS. However, LV myocardial TPSR did not normalize in this time period.

## Introduction

Arterial switch operation (ASO) is the treatment of choice for most neonates with d-transposition of the great arteries (D-TGA).^1^ Nevertheless, the coronary arteries may be at risk of damage during this operation.^2^ Coronary arterial injury can affect the function of the right ventricle and/or left ventricle (LV). The measurement of strain (S) and strain rate (SR) via 2-dimensional speckle-tracking echocardiography (2D STE) is known as a modality for the evaluation of ventricular function.^3^^, ^^4^ There are relatively few studies on the systolic S and SR of the right ventricle using 2D STE in infants and children with simple TGA after ASO.^5^^, ^^6^ Studies on LV systolic S and SR by 2D STE are even fewer.^7^^, ^^8^ The aim of the present study was to evaluate LV myocardial function using systolic longitudinal S and SR measurements by 2D STE in 20 infants with simple TGA and normal ejection fraction after ASO.

## Methods

**Figure 1:**
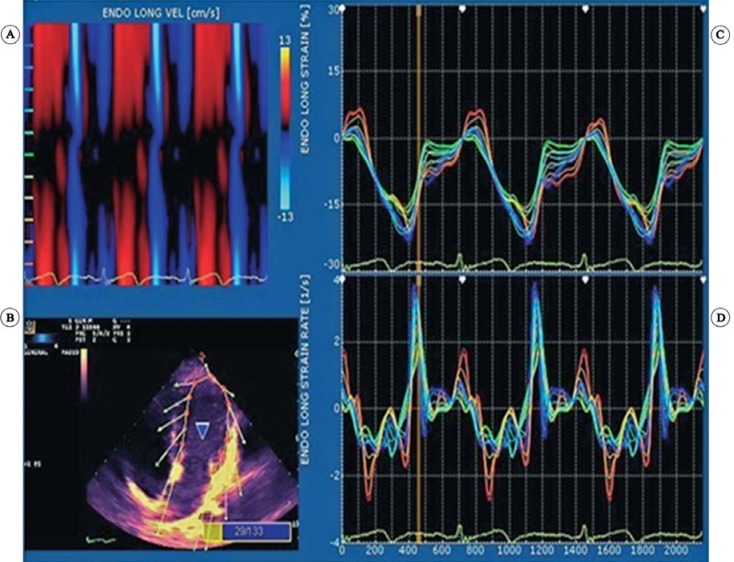
Longitudinal strain and strain rate measurement using X Strain™ software by speckle-tracking two-dimensional echocardiography in the four-chamber view: Figure A shows longitudinal velocity (cm/sec) as a curved anatomical M-mode (CAMM) diagram in three cardiac cycles, Figure B indicates the processing by the software for measuring strain parameter by speckle-tracking of the left ventricular wall. Figures C and D indicate longitudinal strain and strain rate curves of different segments of left ventricle as shown in Figure B.

From March 2011 to December 2012, a total of 87 infants with d-TGA underwent ASO at our center. Asymptomatic patients with simple d-TGA who had the following conditions after ASO were enrolled in this study: time from surgery > 6 months, absence of rhythm abnormality on the electrocardiogram, normal coronary artery pattern at surgery, receiving no medication at the time of study, having no mitral or aortic valve disease or any other structural abnormality, having adequate acoustic window on transthoracic echocardiography, and being operated on by a single surgeon with optimal expertise in ASO at our center. Sixty-seven patients were excluded because they failed to fulfill 1 or more of the above criteria. A control group of age-matched infants and children was also studied. These patients were selected from the infants and children who were referred to the clinic for evaluation of cardiac murmur and were proved to have innocent murmur after a complete echocardiographic examination. Written informed consent was obtained from the parents of both case and control groups. The research protocol was approved by the local ethics committee. The entire study population underwent comprehensive transthoracic echocardiography. Ejection fraction was measured in M-mode images in the standard parasternal long-axis view. LV longitudinal S, SR, time to peak systolic longitudinal strain (TPS), and time to peak systolic longitudinal strain rate (TPSR) were measured in 3 echocardiographic views (i.e., apical 4-, 2-, and 3-chamber views) using XStrain™ installed on a MyLab™ 60 echocardiography machine (Esaote SpA, MyLab 60, Florence, Class 2A). Images were saved in digital format for offline analysis. Echocardiography was performed with a multi-frequency phased-array transducer (PA 122) (3-8 MHz) for patients < 1 year old and a PA230 (1.6-4 MHz) transducer for patients > 1 year old. Gray-scale images were employed with an optimal sector width and a frame rate of 60-90 frames/sec. The tracing of the endocardial border was automatically performed by the software. However, manual correction by the operator was feasible, whenever necessary (Figure 1). Each wall was automatically subdivided into 6 segments. In each view, the 2 opposing walls were comparatively studied. Global peak systolic S for each wall was measured by averaging the values of the segments of that wall and presented as mean ± SD. Global S for each individual patient was measured by averaging the values of the strain values in all 6 opposing walls in 3 views (i.e., 2-, 3-, and 4-chamber views). Based on total cardiopulmonary bypass time, the patients were divided into 2 groups: > 190 minutes and ≤ 190 minutes. They were also subdivided into 2 groups according to aortic cross-clamp time: > 130 minutes and ≤ 130 minutes. Strain values were also compared between patients with a heart rate > 110 beats per minute (bpm) and those with a heart rate ≤ 110 bpm. The data were analyzed using SPSS 18 (SPSS Inc., Chicago II, USA). LV systolic S and SR were reported as mean ± SD, using descriptive statistics. The Kolmogorov–Smirnov test and Shapiro-Wilk test of normality were performed to examine normality for the nominal variables such as sex. ANOVA and the Kruskal-Wallis H-test were used to compare the parameters between the case and control groups. A p value < 0.05 was considered statistically significant. 

## Results

Twenty patients were enrolled. Mean ± SD of age was 15 ± 5 months (minimum: 8 month, maximum: 26 month). Twenty healthy age-matched infants were also examined as the control group. Regarding skewness and kurtosis, our data did not differ from normality significantly. All calculated Z values were between -1.96 and 1.96. In addition, all p values were > 0.05 on the Shapiro-Wilk test. The demographic and general characteristics of the study group are presented in Table 1. 

Global S, TPS, and TPSR were not significantly different between the case group and the healthy infants in the control group. However, global SR was significantly lower in the patients after ASO. 

As is shown in Table 2, segmental comparison of the opposing walls within the case group showed that TPSR was significantly longer in the septal wall in the 4-chamber view than in the lateral wall (p value = 0.005). Moreover, the TPSR of the anterior wall was significantly longer than that of the inferior wall in the 2-chamber view (p value < 0.001). The TPSR of the anteroseptal wall in the 3-chamber view was also longer than that of the posterior wall (p value = 0.031). In the 3-chamber view, peak systolic longitudinal S of the LV anteroseptal wall was significantly lower than that of the posterior wall (-11.57 ± 3.77 vs. -13.49 ± 3.48; p value < 0.001). Longitudinal systolic SR in the septal wall was not significantly lower than that in the lateral wall (p value = 0.028). The values of S, SR, TPS, and TPSR for each of the apical 2-, 3-, and 4-chamber views are depicted in Tables 2, 3, and 4. 

We found no significant correlation between S, SR, TPS, and TPSR between the patients with a heart rate ≤ 110 bpm and those with a heart rate > 110 bpm. There were no positive correlations between LV longitudinal peak systolic S and SR and the variables of heart rate, total cardiopulmonary bypass time, and aortic cross-clamp time.

**Table 1:** General, echocardiographic, and operative characteristics of the study population*

Characteristics	Patients after ASO (n=20)	Healthy Infants in the Control Group (n=20)	F	P value
Age at the time of study (mo)	15.05±5.42	17.13± 9.91	3.104	0 .087
Birth weight (kg)	3.13±0.07	3.16±0.04	0.268	0.704
Age at the time of ASO (d)	12.16±3.60	-	-	-
Weight (kg)	8.83±1.57	10.77±2.03	0.271	0 .606
Systolic blood pressure (mmHg)	94.94±5.86	94.61±6.68	0.109	0 .743
Diastolic blood pressure (mmHg)	49.67±9.51	50.22 ±6.35	0.284	0.597
Heart rate (bpm)	116.39±5.07	114.22±10.24	3.538	0.069
Ejection fraction (%)	62.61±3.39	64.22±4.37	0.669	0.419
Body surface area (m^2^)	0.42±0.06	0.46±0.03	13.597	0.068
Aortic crass-clamp time (min)	127.66±20.82	-	-	-
Total cardiopulmonary bypass (min)	189.16±30.88	-	-	-

ASO, Arterial switch operation

* Data are presented as mean+SD

**Table 2:** Left ventricular longitudinal systolic strain analysis in two-chamber view on echocardiography after arterial switch operation*

Parameters	Patients after ASO (n =20)	Control Group Global (n = 20)	P value
Global			
TPS (msec)	277.46±35.06	288.58±28.73	0.311
S	15.67±11.92	19.87±2.75	0.152
TPSR (msec)	184.98±72.78	176.79±37.85	0.671
SR (s^−1^)	1.40±0.24	5.37±6.08	0.011
Lateral			
TPS (msec)	253.24±61.53	302.75±61.53	0.008
S	12.02±4.03	18.68±3.36	< 0.001
TPSR (s^−1^)	171.22±60.62	187.14±62.54	0.441
SR (s^−1^)	1.38±0.53	8.91±22.71	0.160
Septal			
TPS (msec)	267.42±61.97	288.05±42.95	0.250
S	11.93±3.84	19.03±3.39	0.281
TPSR (msec)	182.55±97.70	179.42±43.72	0.005
SR (s^−1^)	1.12±0.41	6.50±20.47	0.283
Anterior			
TPS (msec)	298.09±75.64	291.11±38.90	0.73
S	31.34±73.45	19.45±4.89	0.511
TPSR (msec)	191.84±137.39	180.77±60.11	< 0.001
SR (s^−1^)	1.48±0.67	5.53±16.13	0.321
Inferior			
TPS (msec)	308.11±150.51	280.07±42.84	0.451
S	13.49±3.48	20.01±4.11	0.001
TPSR (msec)	194.07±118.07	163.12±52.27	0.311
SR (s^−1^)	1.35±0.31	2.42±2.35	0.062
Anteroseptal			
TPS (msec)	271.25±94.58	286.96±48.46	0.534
S	11.57±3.77	20.71±3.08	< 0.001
TPSR (msec)	198.33±71.83	167.59±40.87	0.031
SR (s^−1^)	1.50±0.48	6.88±20.82	0.281
Posterior			
TPS (msec)	266.64±50.97	282.57±52.88	0.361
S	13.68±4.02	21.35±6.17	< 0.001
TPSR (msec)	171.85±70.25	182.72±87.87	0.680
SR (s^−1^)	1.59±0.40	1.99±0.55	0.010

ASO, Arterial switch operation; TPS, Time to peak strain; S, Peak systolic longitudinal strain; TPSR, Time to peak strain rate; SR, Strain rate

* Data are presented as mean+SD

**Table 3:** Left ventricular longitudinal systolic strain analysis in 3-chamber (parasternal long-axis) view echocardiography after arterial switch operation*

Parameters	Basal	Medial	Apical	Global
Anteroseptal wall in the 3-chamber view				
S	-13.18±5.89	-11.44±5.03	-10.08±3.86	-11.57±3.77
SR (s^−1^)	1.89±0.82	1.44±0.49	1.16±0.55	1.50±0.48
TPS (msec)	252.11±114.63	269.66±87.51	292.00±98.00	271.25±94.58
TPSR (msec)	191.22±86.49	193.05±80.70	210.72±102.16	198.33±71.83
Posterior wall in the 3-chamber view				
S	-16.91±5.63	-14.86±4.22	-8.69±2.05	-13.49±3.48
SR (s^−1^)	1.63±0.34	1.47±0.40	0.96±0.38	1.35±0.31
TPS (msec)	298.00±145.58	327.44±240.61	298.88±88.48	308.11±150.51
TPSR (msec)	208.61±138.35	185.50±120.90	188.11±124.28	194.07±118.07

S, Peak systolic longitudinal strain; SR, Strain rate; TPS, Time to peak strain; TPSR, Time to peak strain rate

* Data are presented as mean+SD

**Table 4:** Segmental left ventricular longitudinal systolic strain analysis in 4-chamber view echocardiography after arterial switch operation*

Parameters	Basal	Medial	Apical	Global
Lateral wall in the 4-chamber view				
S	-16.08±4.69	-13.19±4.87	-6.80±3.56	12.02±4.03
SR (s^−1^)	1.82±0.74	1.46±0.57	0.87±0.43	1.38±0.53
TPS (msec)	261.44±73.55	264.38±65.00	233.88±84.96	253.24±61.53
TPSR (msec)	141.22±59.14	152.22±75.68	220.22±138.28	171.22±60.62
Septal wall in the 4-chamber view				
S	-17.03±5.70	-12.71±4.49	-6.04±3.70	11.93±3.84
SR (s^−1^)	1.51±0.53	1.13±0.44	0.72±0.49	1.12±0.41
TPS (msec)	282.50±93.27	270.72±86.97	249.05±96.65	267.42±61.97
TPSR (msec)	184.16±123.56	172.72±136.91	190.77±110.72	182.55±97.70

S, Peak systolic longitudinal strain; SR, Strain rate; TPS, Time to peak strain; TPSR, Time to peak strain rate

* Data are presented as mean+SD

## Discussion

The findings of the present study showed that the normalization of the global systolic SR of the LV myocardium did not occur even 6 to 18 months after a successful ASO in infants with simple TGA. The TPSR of the anterior, septal, and anteroseptal walls and peak systolic S of the anteroseptal wall were still abnormal after successful repair. This indicates that certain segments of the LV myocardium fully recovered later than the others. Furthermore, our findings implied that normal global values did not necessarily indicate that normality of all the segmental components. Therefore, evaluation of the segmental strain parameters, particularly TPSR and peak systolic S, is essential after ASO. We cannot explain the underlying reason for the late normalization of these parameters. However, we would suggest that pathophysiology be investigated at the molecular level of cross-bridging of actin and myosin in the myocytes. Most of the available studies on patients with d-TGA after ASO are on right ventricular systolic S and SR measurements using 2D STE.^5^^, ^^6^ Indeed, there is a dearth of data in the existing literature on LV S and SR using 2D STE following ASO.^7^^, ^^8^ Klitsie et al.^7^ studied on 26 infants with d-TGA who underwent ASO. The authors compared preoperative strain values with postoperative values at the 1st postoperative day, at the time of discharge, and about 1 year after ASO and reported normalization of LV function within the 1st year following ASO. Additionally, total cardiopulmonary bypass time and aortic cross-clamp time in their study were shorter than those in ours (160 ± 33 min and 106 ± 25 min vs. 189.16 ± 30.88 min and 127.66 ± 20.82 min, respectively). Although it can be speculated that total cardiopulmonary bypass time and aortic cross-clamp time may play in a role in the time of recovery of LV function after ASO, we observed no significant differences among the values for S, SR, TPS, and TPSR in the patients with total cardiopulmonary bypass time > 190 minutes and aortic cross-clamp time > 130 minutes and those with total cardiopulmonary bypass time and aortic cross-clamp time less than these values. Of note, the maximum total cardiopulmonary bypass time and aortic cross-clamp time were 250 and 170 minutes, correspondingly, in our study. Interestingly, our results for S and SR in the infants with d-TGA after ASO fell into the normal range, which had been earlier reported by Marcus et al.^9^ for healthy infants. They reported the 5th and 95th percentiles for global longitudinal systolic S to be -18.3 ± 1.9 in the 1st year of age in healthy infants. This may suggest overlapping of values, particularly in the comparison among different ethnic populations. Pettersen et al.^8^ also demonstrated decreased longitudinal systolic S in the late follow-up of their patients after ASO, despite normal ejection fraction. They speculated that several factors such as ischemia, hypoplastic left anterior descending coronary artery, open-chest surgery, and difference in myocardial structure may have led to the decrease in LV longitudinal systolic S. None of our patients had any evidence of ischemia on electrocardiography or any signs of regional wall motion abnormality on 2D echocardiography. Thus, a coronary artery abnormality seems unlikely as a causative factor. Considering the normal ejection fraction, we can speculate that global TPSR is simply a more sensitive variable with later normalization than the other parameters of LV systolic function in patients with simple TGA after ASO. Another hypothesis is to consider this parameter as a subtle marker for those subsets who will later present with decreased exercise capacity.^10^ Accordingly, long-term follow-up of these patients is essential for a clear delineation of the cause of the normalization of the strain parameters and also the exact time at which the strain parameters normalize.

Concerning the limitation of this study, we did not perform serial measurements of S and SR starting from the preoperative period. Evaluation of the serial measurements may be more informative. In addition, we did not measure simultaneous N-terminal prohormone of brain natriuretic peptide (NT-proBNP) levels in our patients to see whether the abnormality in TPSR was associated with any abnormal values of NT-proBNP.

## Conclusion

The results of the current study showed that 6 to 18 months after a successful ASO, the parameters of S, SR, and global TPS were normal in our patients with d-TGA. However, normalization of LV myocardial global longitudinal TPSR did not occur. Our data do not support the notion that total cardiopulmonary bypass time and aortic cross-clamp time, when they are correspondingly less than 250 and 170 minutes, play a role in the duration of time necessary for the completion of the subclinical recovery of LV performance.

## Notes:

***This paper should be cited as:*** Malakan Rad E, Ghandi Y, Kocharian A, Aghayan M. Left Ventricular Function after Arterial Switch Operation as Assessed by Two-Dimensional Speckle-Tracking Echocardiography in Patients with Simple Transposition of the Great Arteries. J Teh Univ Heart Ctr 2016;11(3):105-110.
